# Acute Responses to Resistance Training on Body Composition, Muscular Fitness and Flexibility by Sex and Age in Healthy War Veterans Aged 50–80 Years

**DOI:** 10.3390/nu14163436

**Published:** 2022-08-21

**Authors:** Mario Kasović, Lovro Štefan, Zvonimir Kalčik

**Affiliations:** 1 Department of General and Applied Kinesiology, Faculty of Kinesiology, University of Zagreb, 10 000 Zagreb, Croatia; 2 Division of Sport Motorics and Methodology in Kinanthropology, Faculty of Sports Studies, Masaryk University, 625 00 Brno, Czech Republic; 3 Recruitment and Examination (RECETOX), Faculty of Science, Masaryk University, 625 00 Brno, Czech Republic; 4 Home of Croatian Veterans, 10 000 Zagreb, Croatia

**Keywords:** aging, performance, sex differences, age differences, strength, intervention-induced changes

## Abstract

Background: Although evidence suggests that resistance training should be prescribed as a method to enhance or maintain physical fitness, these findings are mostly based on research on younger men. Studies investigating responses by sex and age to resistance training, especially in war veterans aged ≥50 years, are lacking. Therefore, the main purpose of this study was to examine whether a 4-week resistance training program would have similar effects on body composition, muscular fitness, and flexibility in men and women aged 50–80 years. Methods: Seven-hundred and sixty-four participants were recruited and categorized into two groups each of men and women aged 50–64 and 65–80 years. The training intervention lasted 4 weeks and consisted of three 60 min sessions per week. All participants were tested for each of the following physical fitness components: body composition, push-ups in 30 s, chair-stands in 30 s, sit-ups in 30 s, and a sit-and-reach test. Results: Over the intervention period of 4 weeks, body weight (*p* = 0.002) and the percent of fat mass (*p* < 0.001) decreased, while the percent of lean mass (*p* < 0.001) in push-ups in 30 s (*p* < 0.001), chair-stands in 30 s (*p* < 0.001), sit-ups in 30 s (*p* < 0.001), and sit-and-reach (*p* < 0.001) increased. Significant time*age interactions were shown for push-ups in 30 s (*F_1,763_* = 4.348, *p* = 0.038) and chair-stands in 30 s (*F_1,763_* = 9.552, *p* = 0.002), where men and women aged 50–64 years exhibited larger time-induced changes compared to their older (65–80 yr) counterparts. Effect sizes were similar between sex- and age-specific groups. Conclusions: The 4-week resistance training produced similar pronounced positive effects on body composition, muscular fitness, and flexibility, while men and women aged 50–64 years displayed significantly larger improvements in upper and lower muscular fitness compared with their 65–80-year-old counterparts.

## 1. Background

The prevalence of middle-aged and older adults is expanding worldwide [1]. The process of aging predominantly leads to physiological decline, often accompanied by reductions in lean mass [2,3], power [4], and strength [5]. These declines have been associated with negative health-related outcomes, such as an increased risk of falls [6], hip fractures [7], functional limitations [8], and all-cause mortality [9,10].

Due to structural and physiological losses during aging, previous studies examined efficient methods to increase or even postpone such changes [11]. Studies have documented that resistance training serves as a safe intervention that can increase the level of physical fitness from young to old in both sexes [12,13,14,15,16,17,18]. Furthermore, several studies have shown that resistance training has multiple health-related benefits for overall health [19,20]. However, whether there are sex- and age-specific adaptations to the same training is still unknown [13,18,21].

When considering sex, studies have found that men can increase absolute strength more than women [22,23], although increases in relative strength and hypertension have been similar between the sexes [24,25]. Others have found that women have a greater relative strength increase than men [26,27,28]. The most recent systematic review and meta-analysis showed that men and women adapt to resistance training with similar effect sizes for hypertrophy and lower-body strength, yet women have a larger effect for relative upper-body strength, due to differences in the skeletal muscle level between the sexes [18]. However, well-designed studies with a larger sample size are relatively scare, and sex-related responses to resistance training need to be interpreted with caution.

Age-specific responses to resistance training have been the topic of a few previous studies [12,13,26,29]. Ivey et al. [26] found that young women experience a significantly greater muscle-quality response to resistance training than young men, older men, and older women. In the same study, all groups, except older women, retained resistance-training-induced increases in muscle quality for 31 weeks after the cessation of training [26]. Furthermore, another study demonstrated that young subjects exhibited greater increases in one-repetition maximum compared with the older subjects [12], while Kittilsen et al. [13] found that older adults improved their one-repetition maximum to the same extent as younger groups. 

Findings regarding sex and age responses to resistance training are still inconsistent. Men and women of different age groups have different rates of fatiguability and neuromuscular performance, partially because of differences in anatomy and physiology [30]. Moreover, older individuals may require shorter rest intervals to recover from resistance training, explained by the increase in type I fibers and atrophy of type II fibers occurring with aging [31]. Although resistance training has been recommended for all age groups, its positive effects on neuromuscular impairments and the maintenance of functional performance are especially observed in individuals aged over 50 years [32,33]. By examining sex- and age-specific responses to resistance training, health-related professionals and trainers would be able to plan and program protocols consisting of different training modes, frequencies, and durations to achieve maximum efficiency.

Therefore, the main purpose of the study was to examine whether a 4-week resistance training program would have similar effects on body composition, muscular fitness, and flexibility in middle-aged (50–64 yr) and older (65–80 yr) men and women. We hypothesized that all sex and age groups would have different acute responses to resistance training.

## 2. Methods

### 2.1. Study Participants

Seven hundred and sixty-four middle-aged (range 50–64 yr, mean age ± SD 56.4 ± 5.3 yr) and older (range 65–80 yr, 69.3 ± 4.0) men and women volunteered to participate in this study. All participants went through a rehabilitation center program established by the Ministry of Croatian Veterans (*N* = 2500). The main goal of the center is to improve and enhance the care system and quality of life. The second goal is to reintegrate them into everyday activities, such as social gatherings or sports. The accommodation program usually includes the use of services for up to 30 days. Before entering the study, participants needed to meet the following inclusion criteria: 1) being without chronic diseases, 2) having no psychiatric symptoms or diagnoses, and 3) being without locomotor disabilities (being able to perform exercises with a full range of motion and without pain and discomfort), in order to perform exercises in the4-week intervention program. Of the 2500 participants whose activity was measured, 764 met the inclusion criteria. Such a sample size (*N* = 764) with a two-tailed test, *p*-value set at 0.05, and statistical power of 0.95 would be able to detect a minimum effect size of 0.13. Not all the participants engaged in physical activity in the past 12 months, so the inactive ones were categorized as being sedentary. Prior to the intervention protocol, all participants gave written informed consent for participation, and they all agreed that the data generated from this study could be used for scientific purposes. During the study, the procedures were anonymous and each participant received a unique coded number. We followed the methods of the principles of the Declaration of Helsinki [34], and the Ethical Committee of The Home of War Veterans approved the study (Ethics code number: 2017/4).

### 2.2. Body Composition

Body composition was estimated using bioelectrical impedance analysis (Omron BF500 Body Composition Monitor, Omron Medizintechnik, Hamburg, Germany). The participants needed to stand on metal footpads barefoot and grasp a pair of electrodes fixed on a handle with arms extended in front of the chest [35]. The software used pre-programmed regression equations to predict lean mass and fat mass in percentages. Each participant was instructed not to consume food or water 2 h before the testing procedure. A Seca portable 202 scale (Seca, Hamburg, Germany) and a digital scale (Seca, model 769) were used to measure the standing height (cm) and weight (kg) with a precision of 0.1 cm and 0.1 kg. The body mass index was calculated using the following formula: weight (kg)/height (m^2^).

### 2.3. Muscular Fitness

To assess muscular fitness, three tests were applied: (1) push-ups in 30 s, (2) chair-stands in 30 s, and (3) sit-ups in 30 s. The push-up test measures dynamic muscular endurance [36]. If the participant was not able to regularly perform the test, the test was carried out on the knees, as recommended by previous studies [36]. The final score was recorded as the number of push-ups in 30 s. The chair-stand test estimates lower body strength [37]. The measurer recorded the number of standing up and sitting down sets from a standard chair 40 cm in height, while the participant looked straight ahead with their back in an upright position, legs fully extended and arms folded across the chest. The final score involved counting the number of stand-ups in 30 s. The sit-up test was performed to assess repetitive upper body strength [38]. Before the testing, each participant laid on a soft mat in a supine position, holding the knees bent at an angle of 90° and placing the arms on the chest with the hands on opposite shoulders. The correct attempt included performing a full sit-up to the upright position with elbows touching the thighs and returning to the initial position when shoulders touched the mat surface [38]. The final score was the number of correct sit-ups completed in 30 s.

### 2.4. Flexibility

To assess flexibility, we used the sit-and-reach test [39]. The participant was instructed to reach forward with the arms overlapping along the centimeter tape taped on the floor, while sitting with legs straight under an angle of 90°. The test was measured three times and the best score was recorded in centimeters.

### 2.5. Resistance Training Program

All participants took part in a 4-week resistance training program performed 3 times per week with at least 1 day of rest between each session. All training sessions were supervised by 2 trained exercise specialists who had finished master studies in sports science and had experience in testing of >5 years. Both instructors were familiar with the training exercises and practical lessons given to the participants. The instructors’ role was to supervise the exercises and track the maintenance of intensity. The resistance training was designed to increase lean mass, muscular fitness, and flexibility. Before starting the program, all participants attended two familiarization sessions, as in previous studies [40]. Following familiarization and prior to the intervention, the participants completed the initial one-day testing. Body composition was measured in the morning hours between 9:00 and 11:00 h. Muscular fitness and flexibility were assessed using four tests in the afternoon between 17:00 and 19:00 h. The rest interval between each test was set to 5 min to avoid fatigue. The testing was performed in groups of 15 participants. The same principles were applied to post-intervention testing with similar testing hours (± 1 h) as for the initial testing.

Each resistance training session consisted of a general warm-up period for 5–10 min at moderate intensity, including cycling or performing leg presses. After the warm-up, participants performed 5 different exercises. The exercises included leg presses, knee flexions and knee extensions, horizontal chest presses, seated rows, back pulls, arm curls, and arm extensions. An indirect measurement of the maximum load was calculated, based on the training load at 50% and 60% of the one-repetition maximum. During the first 2 weeks, the participants exercised with ≈50% of the one-repetition maximum in 3 sets (10 repetitions), while in the second 2 weeks, the volume-load increased to ≈60% of the one-repetition maximum in 4 sets (8 repetitions). The pause between the sets was 2 min. All sessions were performed at the same time between 15:00 and 18:00 h with an air-conditioned room temperature of 22 °C and 24 °C. The training sessions were conducted at the same time during the day inside the rehabilitation facility. Two gyms with standardized equipment (machines, free weights, elastic bands, small/big balls, sticks, and mattresses) were used for the testing protocols and training.

### 2.6. Statistical Analysis

Basic descriptive statistics of the study participants are presented as mean and standard deviation. Kolmogorov–Smirnov and Levene’s tests were applied to examine the normality of the distribution and homogeneity of variance for all data before the testing. Within-group changes (initial to final) were tested with the repeated measures ANOVA, while univariate ANOVA was used to calculate between-group differences. If a significant *p*-value was noted, the Bonferroni post hoc test was used to determine the differences among groups. The intervention-induced changes, presented in percent, were analyzed as follows: ((final score-initial score)/initial score). To establish the magnitude of the group difference in body composition, muscular fitness and flexibility, Cohen’s *d* effect sizes were calculated with the following classification: (i) <0.2 (trivial), (ii) 0.2–0.5 (moderate), (iii) 0.5–0.8 (large), and (iv) >0.8 (very large) [41]. Two-sided *p*-values were used, and the significance was set at α < 0.05. All the analyses were calculated in Statistical Packages for Social Sciences v.23 (SPSS, Chicago, IL, USA).

## 3. Results

The number of participants within each sex and age group who undertook the initial and final measurements is given in Table 1.

The basic descriptive statistics of the study participants before and after the 4-week training intervention are presented in Table 2. Over the 4 weeks, significant decreases were observed for body weight (*F_1,763_* = 9.257, *p* = 0.002), and the percentage of fat mass (*F_1,763_* = 63.271, *p* < 0.001), while the increases in the percentage of lean mass (*F_1,763_* = 21.134, *p* < 0.001), push-ups in 30 s (*F_1,763_* = 122.417, *p* < 0.001), chair-stands in 30 s (*F_1,763_* = 200.653, *p* < 0.001), sit-ups in 30 s (*F_1,763_* = 54.627, *p* < 0.001), and sit-and-reach test (*F_1,763_* = 40.172, *p* < 0.001) were obtained. Significant time*age interactions were shown for push-ups in 30 s (*F_1,763_* = 4.348, *p* = 0.038) and chair-stands in 30 s (*F_1,763_* = 9.552, *p* = 0.002), where men and women aged 50–64 years exhibited larger time-induced changes compared with their older (65–80 yr) counterparts. No significant time*sex, time*age or time*sex*age interactions for other variables were noted.

**Figure 1:**
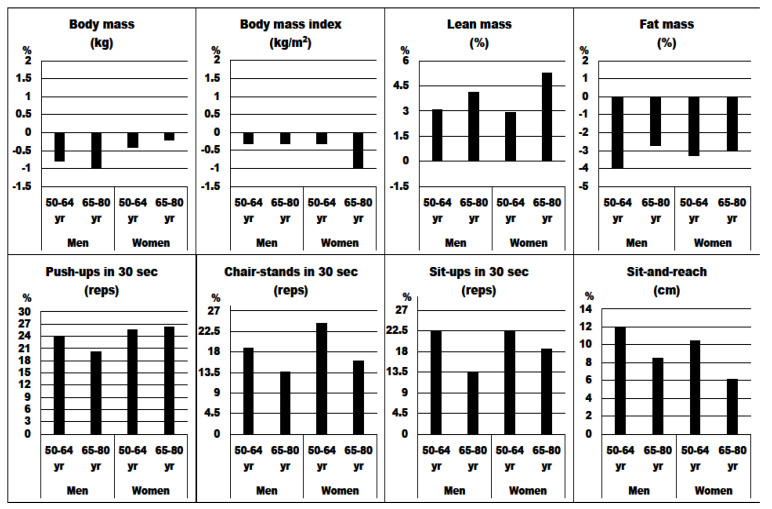
Intervention-induced changes (%) for body composition, muscular fitness, and flexibility before and after the 4-week resistance training across the age groups in years (yr).

**Figure 2:**
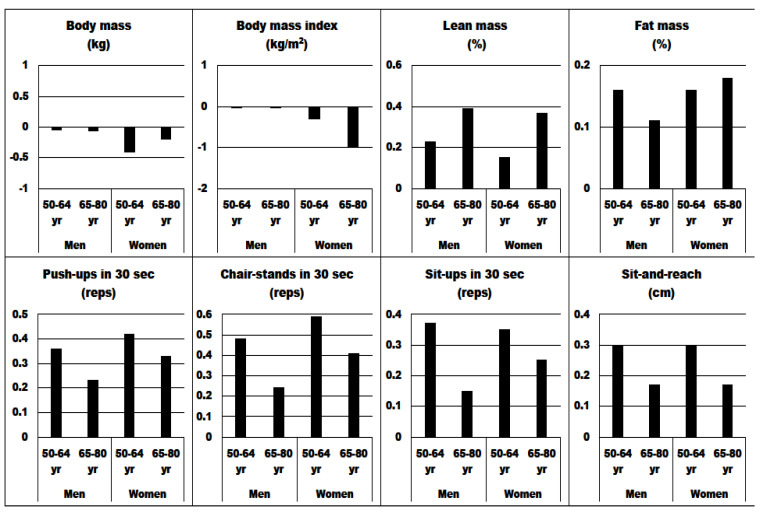
Effect sizes for body composition, muscular fitness and flexibility before and after the 4-week resistance training across the age groups in years (yr).

Figure 1 and Figure 2 show sex- and age-specific intervention-induced changes (%) and effect sizes. After the 4 weeks, women exhibited significantly larger changes for chair-stands in 30 s compared with men in both age groups (24.3% and 16.0% vs. 18.8% and 13.7%, respectively). Additionally, women aged 65–80 years performed better in sit-ups in 30 s (18.6%) than 65–80-year-old men (13.6%). Similarly induced changes in body composition and flexibility in both sex and age groups were observed (*p* > 0.05). The largest effect sizes for muscular fitness were obtained (ranging from 0.15 for sit-ups in 30 s in men aged 65–80 yr to 0.59 for chair-stands in 30 s in women aged 50–64 yr), followed by flexibility (ranging from 0.17 in men and women aged 65–80 yr to 0.30 in men and women aged 50–64 yr). For body composition, the largest effect sizes were observed for the percent of lean mass in men and women aged 65–80 years (0.39 and 0.37, respectively), while for fat mass, the effect sizes ranged from 0.11 in 65–80-year-old men to 0.18 in 65–80-year-old women.

## 4. Discussion

The main purpose of this study was to examine the effects of a 4-week resistance training program on body composition, muscular fitness, and flexibility in middle-aged (50–64 yr) and older (65–80 yr) men and women. The main findings were: (1) significant time changes in all sex and age groups were observed, (2) 50–64 year-old men and women had somewhat larger changes in push-ups in 30 s and chair-stands in 30 s tests compared with their older counterparts, and (3) similar intervention-induced changes (%) and effect sizes between groups occurred.

Studies found that relative upper-body strength increased by 29% in women compared with 17% in men, whereas the increases in lower-body strength were similar [42], which are comparable to our findings in this study. The increases in muscular fitness may be explained by the increases in lean mass and decreases in fat mass, as in previous studies [12]. This statement can be confirmed, because we found significant correlations between lean mass, fat mass, and muscular fitness (*p* < 0.05). Studies showed that different neuromuscular adaptations are one factor contributing to the larger increases in upper-body strength for women, whereas acute recovery following resistance training may be slower in men [18]. Women generally have lower initial levels of physical fitness, causing a ceiling effect for motor skills and better training adaptations compared with men [18]. Although we did not find significant time*sex interactions for any of the study variables, women exhibited larger intervention-induced changes for chair-stands in 30 s. This may be explained by two potential mechanisms. First, women have a greater proportion of type I fibers in the vastus medialis and biceps brachii, indicating better muscular endurance [43,44]. Second, studies showed that men have longer-lasting muscle soreness than women [45]. Most recently, a study by Roberts et al. [18] showed no significant differences between men and women in hypertrophy (effect size = 0.07 ± 0.06, *p* = 0.31) or lower-body strength (effect size = −0.21 ± 0.16, *p* = 0.20); yet, there was a significant effect favoring women for upper-body strength (effect size = −0.60 ± 0.16, *p* = 0.002). Although the changes for push-ups in 30 s were larger for women in our study, unfortunately, we did not collect additional data regarding biological and physiological characteristics, although previous evidence suggests that women tend to have larger strength gains and more muscle growth during the follicular phase [46]. However, our sample size was based on women aged ≥50 yr, and it was reported that the menopausal age starts roughly at the age of 50, dismissing the effects of the menstrual cycle on resistance training [47].

We also found that younger men and women (50–64 yr) had larger effects in push-ups in 30 s and chair-stands in 30 s compared with their older (65–80 yr) counterparts, which is in line with the findings of previous studies [12,48]. Specifically, Lemmer et al. [12] found that younger participants increased their one-repetition maximum strength significantly more than older participants (34% ± 3% vs. 28% ± 3%). Another study showed that young participants had better performance in specific strength for knee extensions and experienced less fatigue across repetitions compared with old participants [48]. Older adults experience physiological changes in terms of age-associated loss of type II fiber number and size, which significantly contribute to the ability to increase strength in response to resistance training [12]. On the other hand, one study reported the same improvements in one-repetition maximum in all age groups from young adults in their twenties and thirties, middle-aged in their forties and fifties and up, to older adults in their sixties and seventies [13]. Along with mechanical and architectural factors associated with the same sex and age responses to resistance training, previous studies acknowledged that the mechanism for similar training-induced increases in muscle quality lies in the increases in motor unit recruitment and decreases in the coactivation of the antagonist muscle groups following training in older adults [26]. Moreover, the ratio between type I and type II fibers may help improve neuromuscular adaptations that enhance the contractile properties of the muscles [26].

Our findings support previous evidence of similar acute responses to resistance training, irrespective of sex and age. Researchers have well-documented that the differences are largely influenced by neural, muscular, and motor learning adaptations [18]. Nevertheless, it is important to understand how resistance training may affect body composition, muscular fitness, and flexibility in men and women of different ages in practice.

This study is not without limitations. First, although we found significant time changes, the 4-week resistance training intervention may have been relatively short compared with those in previous studies with longer study durations (between 6 and 24 weeks) [18]. Second, we did not collect data on the participants’ biological, physiological, or sociodemographic characteristics, which can mediate the resistance training effects. Furthermore, we did not collect data on the level of physical activity prior to the intervention; thus, it is possible that physically active individuals might have had different basal activity compared with untrained individuals. Future research should collect more detailed information regarding the anthropological and health-related status of the participants. In addition to our finding significant changes in physical fitness after the 4-week resistance training, it would be clinically relevant to study the appropriate intervention duration and training mode for producing the largest effects on health-related physical fitness.

## 5. Conclusions

To the best of our knowledge, this is the first study to report marked improvements in body composition, muscular fitness, and flexibility in a large sample of middle-aged and older adults categorized as war veterans, according to sex and age. We found significant changes in all sex and age groups for all variables. Resistance training produced larger improvements in younger men and women in push-ups in 30 s and chair-stands n 30 s compared with older men and women. To conclude, resistance training for 4 weeks increased lean mass, muscular fitness, and flexibility and decreased body weight, body mass index, and fat mass across the groups. Therefore, even a relatively short resistance training intervention may lead to higher physical fitness levels in men and women aged 50–80 years.

## Figures and Tables

**Table 1:** The number of the participants (*N*) with both initial and final measurement, according to sex (men/women) and age in years (yr).

Sex	Men				Women			
Age	50–64 yr		65–80 yr		50–64 yr		65–80 yr	
Study Variables	Initial	Final	Initial	Final	Initial	Final	Initial	Final
Body mass (kg)	271	271	112	112	148	148	52	52
Body mass index (kg∙m^−2^)	265	265	111	111	148	148	52	52
Lean mass (%)	244	244	94	94	127	127	50	50
Fat mass (%)	243	243	93	93	127	127	50	50
Push-ups in 30 s (reps)	198	198	70	70	115	115	24	24
Chair-stands in 30 s (reps)	230	230	82	82	129	129	33	33
Sit-ups in 30 s (reps)	192	192	66	66	115	115	24	24
Sit-and-reach (cm)	229	229	79	79	131	131	32	32

**Table 2:** Body composition, muscular physical fitness, and flexibility for men and women of different ages before and after the 4-week training intervention.

Sex	Men				Women			
Age	50–64 yr		65–80 yr		50–64 yr		65–80 yr	
Study Variables	Initial	Final	Initial	Final	Initial	Final	Initial	Final
Body mass (kg)	95.2 ± 16.5	94.4 ± 17.1	92.9 ± 15.5	92.0 ± 16.8	78.4 ± 15.6	78.1 ± 15.7	80.6 ± 13.2	80.4 ± 13.2
Body mass index (kg∙m^−2^)	29.9 ± 5.0	29.8 ± 5.0	29.8 ± 4.7	29.7 ± 4.8	29.3 ± 5.3	29.2 ± 5.4	30.4 ± 4.4	30.1 ± 4.5
Lean mass (%)	32.5 ± 4.2	33.5 ± 4.6	31.9 ± 3.3	33.2 ± 3.4	27.9 ± 5.4	28.7 ± 5.1	26.4 ± 4.1	27.8 ± 3.4
Fat mass (%)	27.2 ± 6.8	26.1 ± 6.6	26.1 ± 7.0	25.4 ± 6.2	38.7 ± 7.3	37.4 ± 7.6	40.0 ± 6.8	38.8 ± 6.4
Push-ups in 30 s (reps)	11.3 ± 7.0	14.0 ± 7.9	7.4 ± 5.9	8.9 ± 7.2	9.0 ± 5.0	11.3 ± 5.9	7.6 ± 5.3	9.6 ± 6.7
Chair-stands in 30 s (reps)	12.8 ± 4.8	15.2 ± 5.1	10.2 ± 5.4	11.6 ± 6.2	10.3 ± 3.9	12.8 ± 4.6	10.6 ± 3.8	12.3 ± 4.4
Sit-ups in 30 s (reps)	10.3 ± 5.7	12.6 ± 6.7	8.1 ± 7.1	9.2 ± 7.2	8.5 ± 5.1	10.4 ± 5.8	8.6 ± 6.2	10.2 ± 7.0
Sit-and-reach (cm)	40.8 ± 15.9	45.7 ± 16.7	35.5 ± 16.9	38.5 ± 18.5	48.1 ± 15.8	53.1 ± 17.4	45.6 ± 16.6	48.4 ± 16.4

## Data Availability

The datasets used and/or analyzed during the current study are available from the corresponding author on reasonable request.
